# A Reaction-Based Optical Fingerprinting Strategy for the Recognition of Fat-Soluble Samples: Discrimination of Motor Oils

**DOI:** 10.3390/s23187682

**Published:** 2023-09-06

**Authors:** Arseniy A. Pypin, Anna V. Shik, Irina A. Stepanova, Irina A. Doroshenko, Tatyana A. Podrugina, Mikhail K. Beklemishev

**Affiliations:** Department of Chemistry, M.V. Lomonosov Moscow State University, GSP-1, Leninskie Gory 1–3, Moscow 119991, Russia; a_pypin03@mail.ru (A.A.P.); shik.1966@mail.ru (A.V.S.); stepanovamsu@mail.ru (I.A.S.); doroshenkoiran@gmail.com (I.A.D.); podrugina@mail.ru (T.A.P.)

**Keywords:** optical sensing, absorbance, fluorescence, fingerprinting, motor oil recognition, carbocyanine dye oxidation, linear discriminant analysis, k-nearest neighbors algorithm

## Abstract

Optical “fingerprints” are widely used for chemometrics-assisted recognition of samples of different types. An emerging trend in this area is the transition from obtaining “static” spectral data to reactions analyzed over time. Indicator reactions are usually carried out in aqueous solutions; in this study, we developed reactions that proceed in an organic solvent, thereby making it possible to recognize fat-soluble samples. In this capacity, we used 5W40, 10W40, and 5W30 motor oils from four manufacturers, with six samples in total. The procedure involved mixing a dye, sample, and reagents (HNO_3_, HCl, or *tert*-butyl hydroperoxide) in an ethanolic solution in a 96-well plate and measuring absorbance or near-infrared fluorescence intensity every several minutes for 20–55 min. The obtained photographic images were processed by linear discriminant analysis (LDA) and the k-nearest neighbors algorithm (kNN). Discrimination accuracy was evaluated by a validation procedure. A reaction of oxidation of a dye by nitric acid allowed us to recognize all six samples with 100% accuracy for LDA. Merging of data from the four reactions that did not provide complete discrimination ensured an accuracy of 93% for kNN. The newly developed indicator systems have good prospects for the discrimination of other fat-soluble samples. Overall, the results confirm the viability of the kinetics-based discrimination strategy.

## 1. Introduction

Techniques that are based on obtaining spectral or other “fingerprints” of samples with their subsequent chemometric processing allow one to accomplish various classification and discrimination tasks. Essentially, no specific analyte is identified by these methods; rather, a sample is treated as a whole by pattern recognition techniques. Fingerprinting methods are widely used for solving practical problems: e.g., to reveal counterfeits, to identify a manufacturer or type of a sample, to determine freshness of food, and to diagnose a disease by means of blood serum [1,2,3].

Optical fingerprinting can be based on intrinsic absorbance of samples, but more often, it is performed by mixing a sample with a color-forming reagent [4,5,6]. Similarly, fluorescent fingerprinting methods have been refined toward addition of fluorophores to samples [7,8]. A current trend in optical fingerprinting involves another dimension: taking into account a time course of color and fluorescent reactions [9,10,11,12,13,14,15,16]. The use of the kinetic factor makes the fingerprinting technique more powerful. This approach, which we have called “reaction-based fingerprinting”, can help with recognizing samples of very similar composition, for example, with determining the dose received by irradiated foods [11] or recognizing rennet samples [12]. The proposed technique belongs to the family of kinetic assays [17] that are based on an effect of sample components on an indicator reaction rate. The reasons for this effect may differ (e.g., catalysis, binding of the analyte to a metal ion catalyst, or interaction with radical centers of a chain reaction); the actual mechanism is rarely known. It is only important that different components of the sample cause different changes in the color of the indicator system or its fluorescence intensity, thereby enabling a researcher to recognize the samples.

A popular class of dyes suitable for the developing of indicator reactions is carbocyanines, which show not only intense absorbance but also near-infrared (NIR) fluorescence. Carbocyanines can be oxidized, which entails changes in their color along with fluorescence fading; both events can be monitored as a function of time without full-spectrum instrumentation [11,18], thus ensuring the simplicity of the method.

Fats and oils have been successfully recognized by fingerprinting methods based on various techniques: different types of chromatography [19,20,21,22], an electronic nose [23], IR spectroscopy [24,25,26,27], UV-vis absorbance spectra [28], and fluorescence [19,24,29]. Motor oils have been a popular type of samples for the development of recognition and discrimination techniques. They can be recognized by their color [30], by IR spectroscopy [31], and by fluorescence [32,33]. Reaction-based fingerprinting techniques have not yet been applied to fat-soluble samples because of incompatibility of the aqueous-solution-based indicator reactions with the hydrophobic nature of fats and oils.

The purpose of the present work was to devise a methodology for recognizing oil-soluble samples by the reaction-based optical fingerprinting method. The tasks of this study included the choice of redox indicator reactions proceeding in solutions with a relatively low water content (in which oil samples do not form a separate phase) and application of these reactions as indicator processes to the discrimination of six motor oils from four manufacturers. We aimed to develop a technically simple protocol; in particular, we wished to demonstrate the feasibility of using a smartphone camera instead of stationary equipment. One of the tasks was to employ nondiscriminative indicator reactions for recognition purposes by merging their data.

## 2. Materials and Methods

### 2.1. Samples and Reagents

Synthetic motor oils from four manufacturers were provided by a local vendor (Table 1). Discrimination of non-degraded oil samples can be useful for identifying counterfeits and passing off a cheaper oil as higher quality oil. In this study, each of the high-end oils—SRS 5W30, 5W40, and 10W40—has its cheaper analog (LUK, EVE, and GAZ, respectively). Carbocyanine dye **1** (Cy7-hydrazine, CAS No. 2183440-61-9) was purchased from Lumiprobe (Hunt Valley, MD, USA, https://www.lumiprobe.com/, accessed on 1 September 2023), and dye **2** was synthesized by the authors according to published strategies (see Supplementary Materials for the synthetic protocol and spectral data). Ethanol was acquired from Bryntsalov-A (Moscow, Russia) and *t*-butyl hydroperoxide (t-BuOOH) from Sigma (as a 5 M solution in *n*-decane); it was diluted with ethanol (1:9, *v*/*v*) to obtain a 0.5 M working solution. Other reagents were bought from Sigma and used as received.

### 2.2. Equipment

Indicator reactions were carried out in 96-well fluorometric plates (Nunc F96 MicroWell, white, Thermo Scientific, Waltham, MA, USA, cat. No. 136101, or Sovtech, Novosibirsk, Russia, cat. No. M-018). The absorption/reflection of the reaction mixtures in the visible region of the spectrum was photographed with a smartphone camera. NIR fluorescence of the reaction mixtures was monitored using a home-made NIR visualizer [18] containing 11 red LEDs (660 nm) with a power of 3 W as a light source (Minifermer, Moscow, Russia) and a Nikon D80 camera with a light filter cutting off visible light up to 700 nm. The UV-vis spectra were recorded on an SF-102 spectrophotometer (Interfotofizika, Moscow, Russia) in 0.2 × 1.0 cm quartz cells (an internal volume of 0.5 mL and an optical path length of 1 cm). Fluorescence spectra were obtained using a Fluorat-02 Panorama spectrofluorometer (Lumex, St. Petersburg, Russia).

### 2.3. General Procedures

The motor oils were mixed with absolute ethanol in a 1:29 (*v*/*v*) ratio and stored for a day, after which a minor residue formed, which was later discarded. The obtained ethanolic solutions served as oil samples throughout the study. A 1 M solution of HCl in ethanol was prepared by mixing a concentrated aqueous solution of HCl (10 M) with absolute ethanol (1:9, *v*/*v*). To prepare aqua regia as an oxidant, a 1:15 mixture (*v*/*v*) of concentrated aqueous solutions of HCl (10 M) and HNO_3_ (70%, *w*/*w*) was prepared and stored for 24 h before use. Copper(II) solutions were obtained from a 1 M CuSO_4_ aqueous solution by dilution with absolute ethanol. Subsequently, 0.5 M solutions of *t*-BuOOH were prepared from its 5 M solution in *n*-decane by dilution with absolute ethanol.

The indicator reactions were carried out one at a time in 96-well plates. To conduct a reaction, each oil sample was added into six wells (six technical replicates), which gave 36 wells for the six samples (Table 1). All other reactants were added by pipetting in the sequence shown in Table 2 (8-channel pipettes were utilized). The moment of addition of the last reactant was designated as the reaction start. Afterwards, two kinds of images of each plate were captured: (1) visible-light photographs to monitor light absorption and reflection and (2) photographs in the NIR region taken by means of the home-made visualizer to monitor fluorescence intensity under red-light excitation. Seven images of each indicator reaction were captured throughout the reaction (the period during which we could observe the signal changes). The average time interval between the images was the reaction duration (min) divided by 7. Given that the reaction duration varied from 7 min (reaction of dye **1** with aqua regia) to nearly 1 h (reaction of dye **2** with *t*-BuOOH), the images had to be captured every minute in the former case and every 8 min in the latter case. The number of images taken for each indicator reaction had to be minimized for the sake of simplicity and maximized in terms of accuracy. However, we found that the number of images used was sufficient to obtain 100% accuracy values, for which reason the employed data size was considered appropriate.

Visible-light absorption/reflection images were captured under ambient light to simplify the experimental protocol and to demonstrate that smartphone images are suitable for solving discrimination problems. The 96-well plate was positioned on the benchtop and photographed from a distance allowing us to visualize the whole plate; later, the image was cropped to obtain a 6 × 6-well region of interest. The NIR fluorescence images, which required 660 nm LED excitation, were captured in the visualizer cabinet from a 35 cm distance.

The size of a smartphone image corresponding to the 36-well portion of the plate was ~800 × 800 pixels (640 kB); an image from the NIR visualizer was 1100 × 1100 pixels (1.2 MB).

### 2.4. Data Processing

The photographic images were digitized in ImageJ ver. 2.0.0-rc-61/1.51n software (Fiji). RGB splitting was performed to obtain red, green, and blue channel intensities (R, G, B) for the visible-light photographs; for monochrome NIR fluorescence images, only the overall black-and-white intensity was quantified. A round spot was manually selected in the central part of each well (the diameter of the spot was 1/3 of the well diameter) to determine the mean intensity across the spot. The resulting intensities (that varied within 0–255) were organized in data tables, where columns represented different channels (RGB visible and NIR) quantified at different reaction time points (28 columns); the rows of the table represented the six oil samples analyzed six times each, which gave 36 rows of data (observations). Table S1 is an example of a data table. If some of the images showed no difference between samples, these data were excluded from further processing.

The data organized in the above way were subjected to linear discriminant analysis (LDA) or to the k-nearest neighbors algorithm (kNN) using the XLSTAT add-on for Microsoft Excel 2016 (ver. 2016.02.28451, Addinsoft, New York, NY, USA). The following settings for LDA were found to be the most appropriate: within-class covariance matrices were assumed to be equal; the stepwise (backward) model selection was activated with the threshold value of 0.05 to include the variable and 0.10 to leave it out. The settings of kNN included the use of the Euclidean type of distances and automatic determination of the number of neighbors based on cross-validation (the number of neighbors usually varied between 3 and 5). A significance level of 5% was chosen in the study.

LDA and kNN are supervised techniques in which the software is informed about the assignment of the observations to classes (in this work, there were six classes corresponding to six oils). The accuracy of discrimination of the samples was assessed by a validation procedure. The observations were divided into a training set (83% of data) and a validation set (17% of data); the latter was not used for constructing a model. The validation set of six observations was randomly selected at each run of XLSTAT (for example, two observations from the 1st oil, one from the 2nd oil, none from the 3rd oil, and so forth), and the LDA model was constructed by means of the remaining 30 observations. In accordance with the model, the software assigned each tested observation to one of the six classes. As a result of such assignment, a confusion matrix was built (Table S2 in Supplementary Materials), where a relationship between the true class and predicted class could be seen for each observation. Percent accuracy of discrimination was then calculated as the number of correctly assigned observations divided by the total number of observations in the validation set. The LDA procedure was performed no fewer than five times on the dataset, and the obtained values of accuracy were averaged to arrive at the mean value of accuracy, which served as a characteristic of the dataset (one of the indicator reactions or their combination).

For kNN, the validation protocol was similar, with the difference being that no automatic random selection of a validation set was available in the software; instead, we manually moved one of the six observations of each sample to the validation set. For example, to compile the first validation set, we removed the first observation of the 1st sample, the second observation of the 2nd sample, and so forth until the 6th observation of the 6th sample. To compile another validation set, we removed the second observation of the 1st sample, the third observation of the 2nd sample, and so forth. The compiled validation sets were each used in an independent run of the software to determine discrimination accuracy.

## 3. Results and Discussion

### 3.1. The Choice of Indicator Reactions

**Scheme 1:**
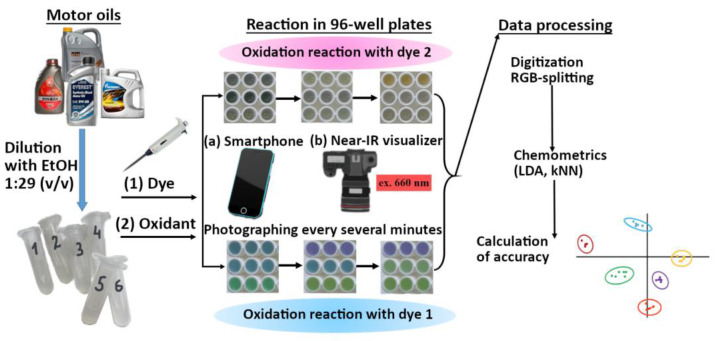

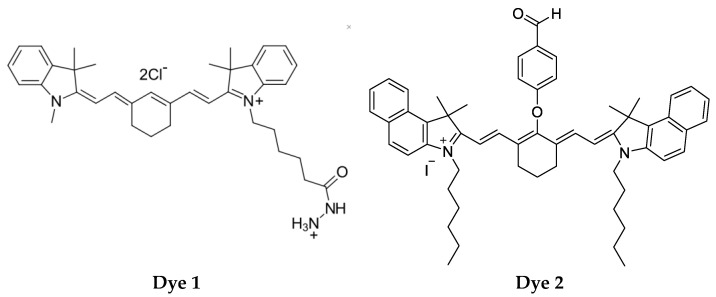
Principles of the proposed method and structures of the carbocyanine dyes.

The principle of the proposed method is illustrated in Scheme 1. The ethanolic solutions of motor oil samples were mixed with components of an indicator reaction in a 96-well plate, and products of the reaction were monitored (Section 2.3). Out of the vast variety of possible indicator reactions, redox-type processes, such as the oxidation of dyes with peroxides or peracids, have proven to be efficient in recognition tasks involving reaction-based methods [9,10,11,12]. Carbocyanine dyes have been successfully used for these purposes because changes in both their absorbance and fluorescence can be measured. We noticed that carbocyanine dyes with reducing side groups can be oxidized under milder conditions than dyes without such groups can. A possible reason is formation of active radicals during the oxidation of the side groups; these radicals can subsequently initiate the oxidation of the polymethine chain of the carbocyanine dye, resulting in its color change and fluorescence fading. The structures of carbocyanine dyes with reducing side groups used in this study are presented in Scheme 1.

The task of developing new indicator processes involved the conducting of a redox reaction in a medium with a sufficiently low water content, in which the oil components would remain in solution. A number of oxidants were tested for the oxidation of dyes **1** and **2** in ethanol: fat-soluble (*tert*-butyl hydroperoxide), water-soluble (nitric acid and the mixture of HCl and HNO_3_: aqua regia), and atmospheric oxygen (the list of the indicator reactions is given in Table 2). All reactants were added in the form of ethanolic solutions. For most reactions, water was supplied only with the 1 M ethanolic solution of HCl and/or Cu^2+^ solution, which amounted to less than 0.7 mg of water per well. More water was introduced with aqueous aqua regia (up to 20 µL/well) and concentrated HCl for the reaction of dye **1** with oxygen (40 µL/well), but it still did not cause phase separation.

**Figure 1:**
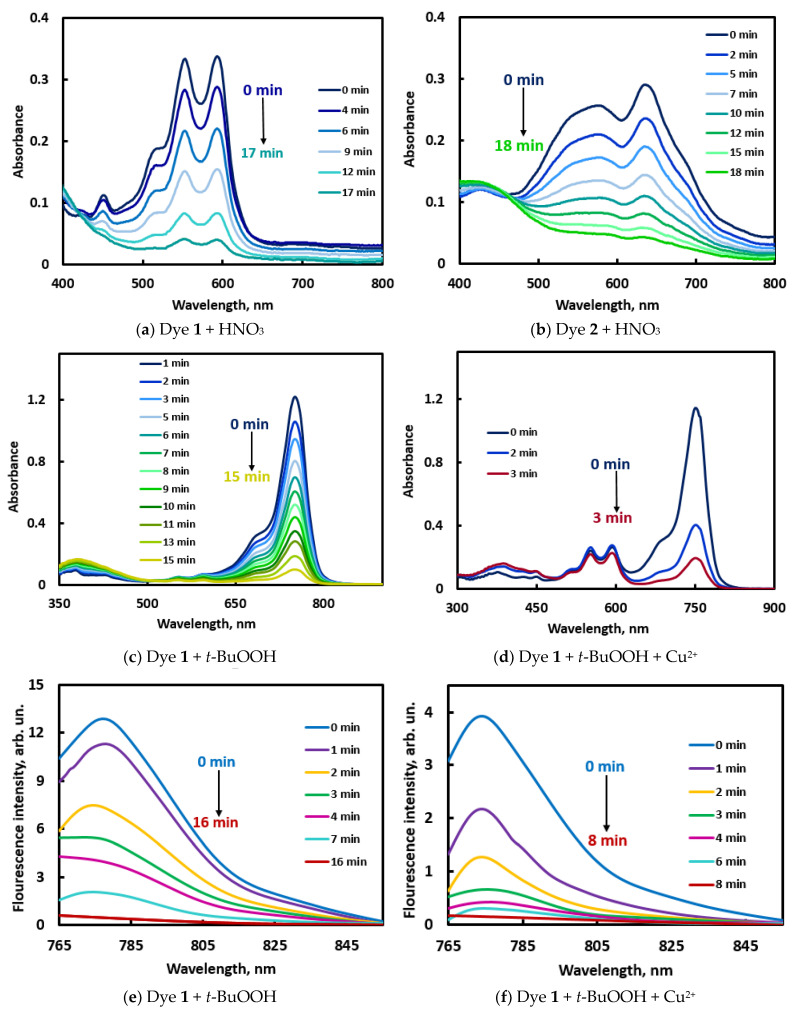
Absorbance (**a**–**d**) and NIR fluorescence (**e**,**f**) spectra at different reaction time points for the indicator reactions named under the images. Emission (**e**,**f**) was excited at 750 nm. Amounts of reactants were as follows: (**a**) 20 µL of 0.1 g/L dye **1**, 500 µL of EtOH, 60 µL of concentrated HNO_3_; (**b**) 20 µL of 0.3 g/L dye **2**, 500 µL of EtOH, 60 µL of concentrated HNO_3_; (**c**) 10 µL of 0.1 g/L dye **1**, 380 µL of EtOH, 20 µL of 1 M HCl, 30 µL of 0.5 M *t*-BuOOH; (**d**) 10 µL of 0.1 g/L dye **1**, 380 µL of EtOH, 20 µL of 1 M HCl, 10 µL 1 × 10^−4^ М Cu^2+^, 30 µL of 0.5 M *t*-BuOOH; (**e**) 10 µL of 0.1 g/L dye **1**, 10 µL of 1 M HCl, 280 µL of EtOH, 10 µL of 0.05 M *t*-BuOOH; (**f**) 10 µL of 0.1 g/L dye **1**, 10 µL of 1 M HCl, 260 µL of EtOH, 10 µL 1 × 10^−4^ М Cu^2+^, 10 µL of 0.05 M *t*-BuOOH. All dilutions were performed by means of absolute ethanol (EtOH).

In preliminary experiments, amounts of reactants were selected so that reaction rates could be conveniently monitored (measurement duration had to be ≤1 h). To examine the time course of the reactions, the solutions were placed in quartz cells for the registration of a full spectrum. Both dyes manifested spectral changes under the action of oxidizing agents: the main absorption bands became less intense with time and approached zero within 7–40 min (Figure 1 and Figure S1 in Supplementary Materials). Introduction of copper(II) into the systems containing *t*-BuOOH accelerated the oxidation of dyes (Figure 1d,f vs. Figure 1c,e), sometimes along with a spectral shift (Figure 1c vs. Figure 1d). NIR fluorescence intensities were taken into account only for the reactions with *t*-BuOOH (Figure 1e,f) but not with HNO_3_. For practical applications, the duration of measurement should be neither too long (to prevent the method from becoming time-consuming) nor too short (it should be much longer than the period before the addition of the last component to all samples in the 96-well plate). The reaction rates observed in the studied systems were assumed to be applicable to a reaction in the presence of a motor oil.

In some systems, the time point of color change (Figure 1c,d) did not match the time required for complete fluorescence fading under the same conditions (Figure 1e,f). This phenomenon could be explained by the formation of different products of partial oxidation, each of which has its own absorbance and fluorescence spectrum and its individual rate of further oxidation. Such complexity of oxidation reactions may help with discrimination tasks if absorbance and fluorescence intensities serve as independent variables.

The full spectra were obtained only to study the indicator reactions, whereas further work with oils was carried out using the photographic principle of signal registration.

### 3.2. Indicator Reactions in the Presence of Oil Samples

**Figure 2:**
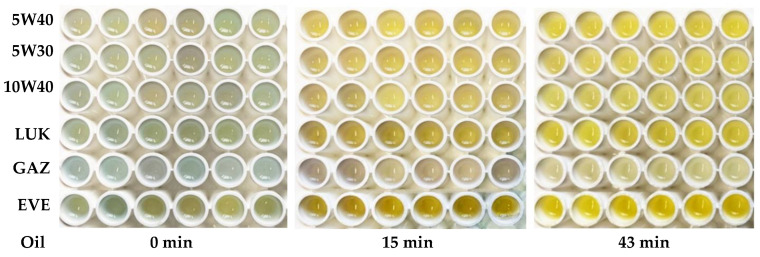
Visible-light images of reacting system “*oil + dye **1** + HNO_3_*”, as obtained with the help of a smartphone camera. Replicate experiments for a given sample are in a row. Time after the reaction start is shown under each image.

The key feature of the indicator process in reaction-based fingerprinting is its ability to change its rate in the presence of an assayed sample. We tested the proposed reactions with the motor oil samples in 96-well plates according to the protocol given in Table 2. Six parallel runs were performed for each sample. Examples of the resultant images are presented in Figure 2 and Figure S2. One can see that recognition of samples by eye was difficult, and subsequent chemometrics-assisted processing of the images was necessary.

The photographs of the reaction mixtures in the wells were digitized (a round part of an image without a glare was selected) and then subjected to RGB splitting. For constructing kinetic curves for each reaction, a channel most promising in terms of recognition of samples was chosen. For example, for the *dye **1** + HNO_3_* reaction (Figure S3), initial (at zero reaction time) RGB intensities differed between samples due to differences in their natural color; in the course of the reaction, R and G channel intensities increased with time, whereas no regular trend was seen for the B channel; however, the latter channel was the most discriminative due to higher repeatability and noticeable differences between oil samples. At the maximum duration, the sample signals varied between 68 and 124 units (56 units) for channel B, whereas this value varied within 158–174 (16 units) and 144–159 (15 units) for R and G channels, respectively. For these reasons, the B channel was chosen for processing.

**Figure 3:**
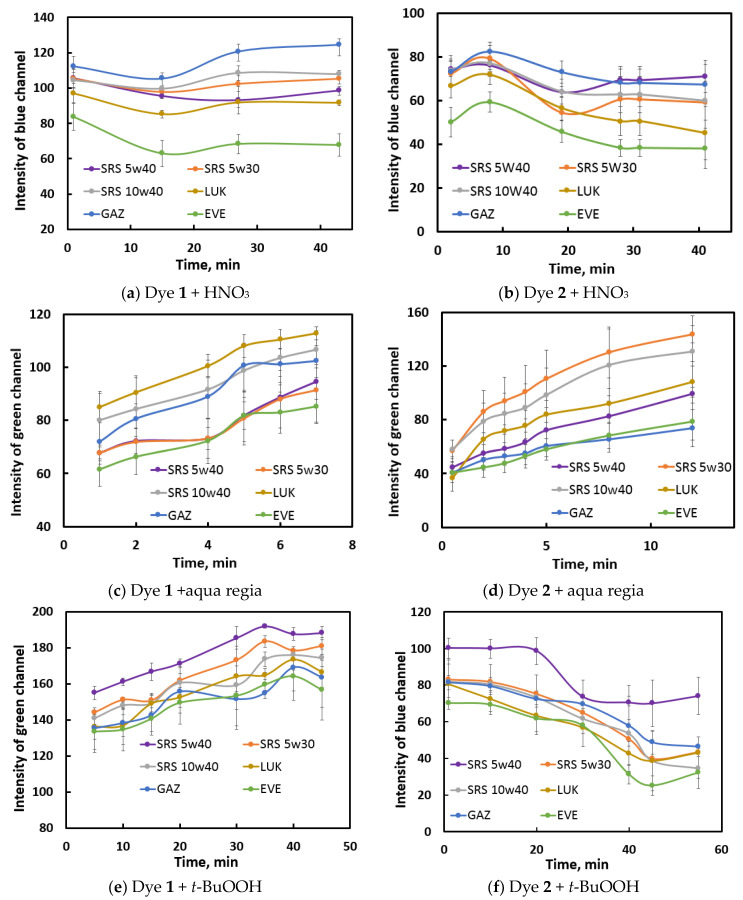
Kinetic curves of indicator reactions in the presence of oil samples shown below each graph. Each curve is a result of averaging of six replicate runs, and the error bars denote standard deviations. The vertical axis is the intensity of a color channel of the images obtained from photographs. See Table 2 for the reaction conditions.

Similar work was carried out with the other indicator reactions; the selected channels are shown in Figure 3. For NIR fluorescence, no selection was necessary because it is a black-and-white signal. For the selected channels, the results of the six replicate measurements were averaged, and the resulting kinetic curves were constructed (Figure 3 for visible-light photographs and Figure S4 for NIR images).

It can be deduced from the obtained curves that oils exert different effects on the kinetic-curve shape: EVE and GAZ oils do so in the reactions with HNO_3_ as the oxidant; the SRS 5W40 oil and sometimes SRS 5W30 oil do so in the reactions with *t*-BuOOH as the oxidant. For the aqua regia reactions, the oils tended to form groups of 2–3 samples. More meticulous discrimination could be achieved after data processing.

### 3.3. Discrimination of Oils by Individual Indicator Reactions

The data tables for the chemometric data processing included intensities of all color channels for the six parallel observations of six samples; the columns of the tables represented different reaction time points for an individual indicator reaction. The accuracy of discrimination of oils by LDA and kNN was verified by the validation procedure; percent accuracy was calculated as the ratio of correctly assigned observations to the total number of observations (6) in the validation set, and the results are presented in Table 3.

For kNN, no indicator reaction was found to completely discriminate the six oil samples, even when all available color channels were employed (and NIR fluorescence data, if any). The best in terms of accuracy was reaction 5 (dye **1** + HNO_3_, 90%). Attempts to reduce the amount of kNN-processed data by using only one color channel (as selected in Section 3.2) yielded the same (for reactions 1 and 3) or lower accuracy (for the other reactions), though for the best reaction, it remained as high as 87% (Table 3).

For the LDA-processed data, the two dyes showed a similar trend: catalytic oxidation of a dye by *t*-BuOOH was the least efficient, and aqua regia as the oxidant ensured results close to those of *t*-BuOOH; accuracy improved when *t*-BuOOH was used without Cu^2+^ and aerial oxidation of dye **1** was performed. The most efficient were the reactions with nitric acid as the oxidant: all six samples were discriminated by reaction 5 (100% accuracy for LDA). A similar reaction, oxidation of dye **2** by HNO_3_, also gave a good result (93% accuracy for LDA), which was not the case with kNN processing.

**Figure 4:**
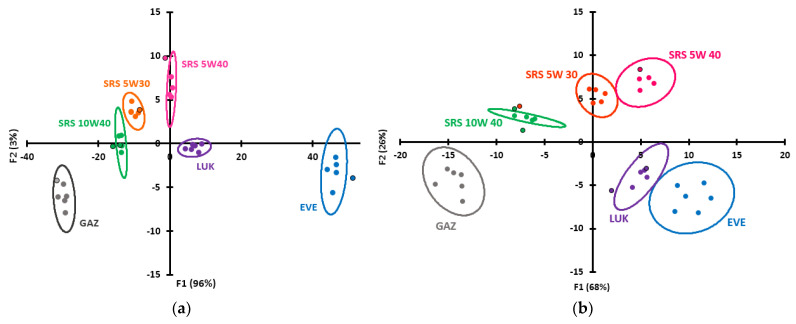
Examples of LDA score plots for the indicator reactions: (**a**) dye **1** + HNO_3_; (**b**) dye **1** + O_2_. Validation points are shown as circles having a black border.

LDA score plots (Figure 4) were constructed to visualize the discrimination of the training samples (highlighted by ellipses) and validation samples. For example, in graph b, readers can see that one of the SRS 5W30 validation points was located close to the SRS 10W40 group and was assigned incorrectly. Other validation points were located close to their correct classes.

It is noteworthy that complete discrimination was achieved only when the kinetic curves were analyzed. If only a single photograph was used, then the recognition was poor. For instance, processing of the zero-time visible image for the most efficient reaction “*dye **1** + HNO_3_*” (for all three color channels) yielded an LDA discrimination accuracy of 68% (averaged from 10 consecutive LDA runs). Similarly, for a 2 min photograph of the *dye **2** + HNO_3_* reaction, an accuracy of 40% was achieved. Both values are substantially lower than those listed in Table 3. Kinetic data improve discrimination because they characterize samples in more detail.

Overall, there was at least one system allowing for complete discrimination of all six samples (oxidation of dye **1** by HNO_3_ in combination with processing of all RGB color channels by LDA).

### 3.4. Discrimination of Oils by Combining Several Reactions

As demonstrated above, most of the individual indicator reactions were not sufficiently discriminative (accuracy was well below 100%, Table 3). It was necessary to improve discrimination accuracy and render these data useful for recognition purposes. Six reactions giving the lowest accuracy (1, 2, 3, 6, 7, and 9) were selected for this purpose. All the results were merged to obtain one data table (179 columns) and were processed by kNN or LDA (Table 4). Moreover, smaller reaction sets were also studied: the reactions with the lowest individual accuracy for kNN (not exceeding 73%, Table 3) were removed one by one. The results revealed that LDA performed best in individual reactions (Section 3.3), but this was not the case for the merged data: the accuracy of discrimination was as low as 60% and did not increase with a decrease in the number of reactions. By contrast, kNN not only showed superior accuracy (87% for the initial dataset) but also manifested a maximum of accuracy (93%) for the shortened dataset of only four reactions (2, 3, 6, and 9). Because we removed the reactions with the lowest accuracy, this action can be regarded as removal of noise and thus may improve the results. In addition, kNN is a completely nonparametric approach: no assumptions are made about the shape of the decision boundary. This property may be an advantage for very complicated datasets. In the case of LDA, we are limited to only linear discrimination functions.

Overall, if there are a number of indicator reactions and each of them does not allow one to solve a discrimination problem, then data merging and selecting appropriate reactions can improve accuracy (for example, from 73% for individual reaction 2 to 93% for the set of four reactions). It should be mentioned that all the accuracy values reported above pertain to single observations, whereas all samples are measured in several replicates. If the accuracy of a single observation is 93% and the number of parallel observations for each sample is 6, as in this study, then the accuracy of assignment for the whole set of observations for the oil sample will be 99.4% (see Supplementary Materials for the formulas).

## 4. Conclusions

In this study, an innovative optical fingerprinting strategy based on indicator reactions was refined. Indicator reactions proceeding in an organic solvent with a low water content were shown to be applicable to the discrimination of fat-soluble samples. Six motor oils of various types from several manufacturers were used as model samples; they were recognized with 100% accuracy, which confirms the high discriminatory power of the method. The reactions with nitric acid as an oxidant were found to be the most discriminative among several tested redox systems.

The method is rapid and simple, is based on a smartphone camera, and does not involve any full-spectrum instruments. A NIR visualizer is also unnecessary for the most discriminative reactions. Using a smartphone implies that illumination conditions are flexible; the user should only try to capture images at approximately the same light intensity. This approach did not cause any problems with data processing, and its practicality is thus confirmed in this and previous [11] studies, with satisfactory results.

A limitation of the proposed strategy is the necessity to select indicator reactions when switching to a new type of sample. On the other hand, appropriate indicator processes can be found for a wide range of samples of different types, which means that the strategy is rather versatile. Other advantages of the method include the use of standard software for digitizing and processing the obtained photographs and the feasibility of complete discrimination by means of only one indicator reaction with a commercial dye. The data from several other reactions (each of which alone is nondiscriminative) can be combined to improve discrimination accuracy. The strategy has good prospects for the recognition of other oils and fats.

## Figures and Tables

**Table 1:** Motor oil samples.

Abbreviation	Name	SAE Grade	Manufacturer
SRS 5W30	Cargolub TFX	5W30	SRS Schmierstoff Vertrieb GmbH ^1^
SRS 5W40	Cargolub TFX	5W40	SRS Schmierstoff Vertrieb GmbH ^1^
SRS 10W40	Cargolub TFX	10W40	SRS Schmierstoff Vertrieb GmbH ^1^
LUK	Genesis Armortech	5W30	Lukoil (LLK International) ^2^
EVE	Everest	5W40	US Global Petroleum ^3^
GAZ	Gazpromneft Premium	10W40	Gazpromneft-S ^2^

Note. ^1^ Salzbergen, Germany; ^2^ Moscow, Russia; ^3^ Franklin Park, IL, United States.

**Table 2:** Protocols of indicator reactions with motor oil samples in 96-well plates.

Dye	Oxidant	Solutions Added into a Well (in the Order Shown)
**1**	*t*-BuOOH	(a) 10 µL of motor oil solution (MO); (b) 10 µL of dye **1**; (c) 10 µL of 1 М HCl; (d) 180 µL of EtOH; (e) 90 µL of *t*-BuOOH
	*t*-BuOOH (Cu^2+^ as catalyst)	(а) 10 µL of MO; (b) 10 µL of dye **1**; (c) 10 µL of 1 М HCl; (d) 200 µL of EtOH; (e) 10 µL of 10^−4^ М Cu^2+^; (f) 60 µL of *t*-BuOOH
	HNO_3_	(а) 10 µL of MO; (b) 10 µL of dye **1**; (c) 250 µL of EtOH; (d) 30 µL of concentrated HNO_3_
	Aqua regia	(a) 10 µL of MO; (b) 10 µL of aqua regia; (c) 270 µL of EtOH; (d) 10 µL of dye **1**
	O_2_	(а) 10 µL of MO; (b) 10 µL of dye **1**; (c) 60 µL of concentrated HCl; (d) 220 µL of EtOH
**2**	*t*-BuOOH	(a) 10 µL of MO; (b) 10 µL of dye **2**; (c) 10 µL of 1 М HCl; (d) 180 µL of EtOH; (e) 90 µL of *t*-BuOOH
	*t*-BuOOH (Cu^2+^ as catalyst)	(а) 10 µL of MO; (b) 10 µL of dye **2**; (c) 10 µL of 1 М HCl; (d) 220 µL of EtOH; (e) 20 µL of 10^−4^ М Cu^2+^; (f) 30 µL of *t*-BuOOH
	HNO_3_	(а) 10 µL of MO; (b) 10 µL of dye **2**; (c) 250 µL of EtOH; (d) 30 µL of concentrated HNO_3_
	Aqua regia	(a) 30 µL of MO; (b) 30 µL of aqua regia; (c) 230 µL of EtOH; (d) 10 µL of dye **2**

Notes. Concentration of dyes **1** and **2**: 0.1 g/L, concentration of *t*-BuOOH: 0.5 g/L. Abbreviations: EtOH, absolute ethanol; *t*-BuOOH, *tert*-butyl hydroperoxide; MO, motor oil solution. All solutions were prepared using EtOH.

**Table 3:** Discrimination of six motor oil samples by LDA or kNN using individual indicator reactions.

Dye	Oxidant	Reaction Number	Type of Data Used *	Discrimination Accuracy **, %		
				kNN (All Channels)	kNN (1 Channel ^#^)	LDA (All Channels)
**1**	*t*-BuOOH (Cu^2+^ as catalyst)	1	Vis. + NIR	33	33 (NIR)	40
	Aqua regia	2	Vis.	73	60 (G)	50
	*t*-BuOOH	3	Vis. + NIR	57	57 (G)	67
	O_2_	4	Vis.	40	30 (G)	70
	HNO_3_	5	Vis.	90	87 (B)	100
**2**	*t*-BuOOH (Cu^2+^ as catalyst)	6	Vis. + NIR	67	57 (NIR)	50
	Aqua regia	7	Vis.	46	37 (G)	57
	*t*-BuOOH	8	Vis. + NIR	63	43 (B)	73
	HNO_3_	9	Vis.	53	57 (B)	93

* Vis.: photographs captured with the smartphone camera; NIR: photographs obtained in the NIR visualizer. ** From the validation set of six observations, averaged for five independent runs of the software. ^#^ The channel selected in Section 3.2 (shown in parentheses).

**Table 4:** The results of merging of data from nondiscriminative reactions: accuracy of discrimination of six oil samples (from five various validation sets; all available color channels were used).

Total Number of Reactions	Reactions * Used for Merging the Data	Discrimination Accuracy **, %	
		for kNN	for LDA
6	1, 2, 3, 6, 7, 9	87	60
5	2, 3, 6, 7, 9	76	47
5	1, 2, 3, 6, 9	80	53
4	2, 3, 6, 9	93	33
3	2, 3, 6	69	33
2	2, 6	63	23

* See Table 3 for the numbering. ** Estimated using a validation set as in Table 3.

## Data Availability

All data necessary to reproduce our results are presented in the main text and in the electronic Supplementary Materials. Raw data are available upon request.

## Supplementary Materials

The following supporting information can be downloaded at https://www.mdpi.com/article/10.3390/s23187682/s1: Synthesis of dye 2 (protocols and spectral data); Table S1: An example of a data table containing intensities of photographic images of samples; Table S2: An example of a confusion matrix created by the XLSTAT LDA software for evaluating the accuracy of discrimination by a validation procedure; Figure S1: Absorbance spectra at different reaction time points for the indicator reactions named above the images; Figure S2: Visible-light images of the reacting system “*dye **2** + HNO_3_*”, as captured by a smartphone camera; Figure S3: Kinetic curves for the reaction between dye 1 and HNO_3_, as plotted for three color channels (R, G, and B); Figure S4: Kinetic curves of indicator reactions (a) Dye **1** + *t*-BuOOH and (b) Dye **2** + *t*-BuOOH + Cu^2+^ in the presence of oil samples; accuracy of assignment of an oil sample as a whole (six observations) [34].
