# Supplementary material for: A Reaction-Based Optical Fingerprinting Strategy for the Recognition of Fat-Soluble Samples: Discrimination of Motor Oils
Source: Sensors (Basel). 2023 Sep 6;23(18):7682. doi: 10.3390/s23187682 (PMC10535383; doi:10.3390/s23187682)
Supplement: Supplementary file 1 [file sensors-23-07682-s001.zip › sensors-2556968-supplementary.pdf]

Electronic Supplementary Information to paper

# A Reaction-Based Optical Fingerprinting Strategy for the Recognition of Fat-Soluble Samples: Discrimination of Motor Oils

by Arseny A. Pypin, Anna V. Shik, Irina A. Stepanova, Irina A. Doroshenko, Tatyana A. Podrugina, Mikhail K. Beklemishev*

## Contents of the ESI

- **Synthesis of dye 2** (protocols and spectral data)
- **Table S1.** An example of a data table containing intensities of photographic images of samples.
- **Table S2**. An example of a confusion matrix created by the XLSTAT LDA software for evaluating the accuracy of discrimination by a validation procedure.
- **Figure S1.** Absorbance spectra at different reaction time points for the indicator reactions named above the images.
- **Figure S2.** Visible-light images of the reacting system *dye **2** + $HNO_3$*, as captured by a smartphone camera.
- **Figure S3.** Kinetic curves for the reaction between dye **1** and $HNO_3$, as plotted for three color channels (R, G, and B).
- **Figure S4.** Kinetic curves of indicator reactions (a) Dye **1** + t-BuOOH and (b) Dye **2** + t-BuOOH + $Cu^{2+}$ in the presence of oil samples.
- **Accuracy of assignment of an oil sample as a whole (six observations)**
- **References**

**Synthesis of dye 2** (2-((E)-2-((E)-2-(4-formylphenoxy)-3-((E)-2-(3-hexyl-1,1-dimethyl-1,3-dihydro-2H-benzo[e]indol-2-ylidene)ethylidene)cyclohex-1-en-1-yl)vinyl)-3-hexyl-1,1-dimethyl-1H-benzo[e]indol-3-ium iodide)

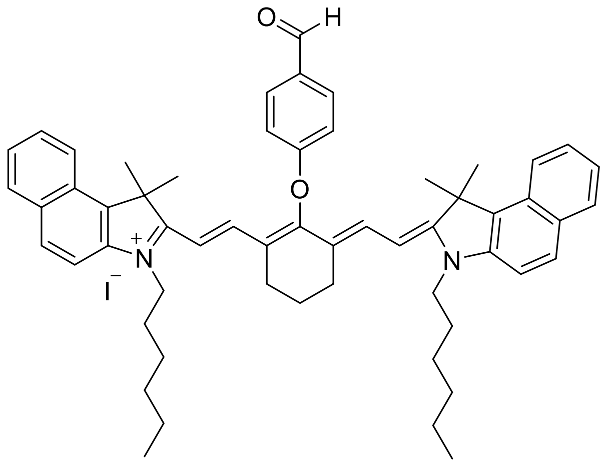

**2**

Compound **I** was obtained as described in [1]. Compounds **II** and **III** (dye **2**) were synthesized as shown in the Scheme and described below in more detail:

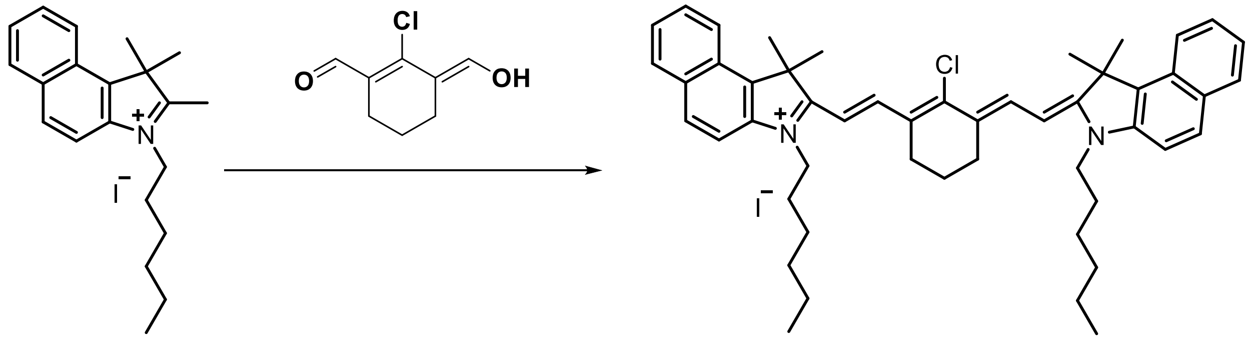

**I** **II**

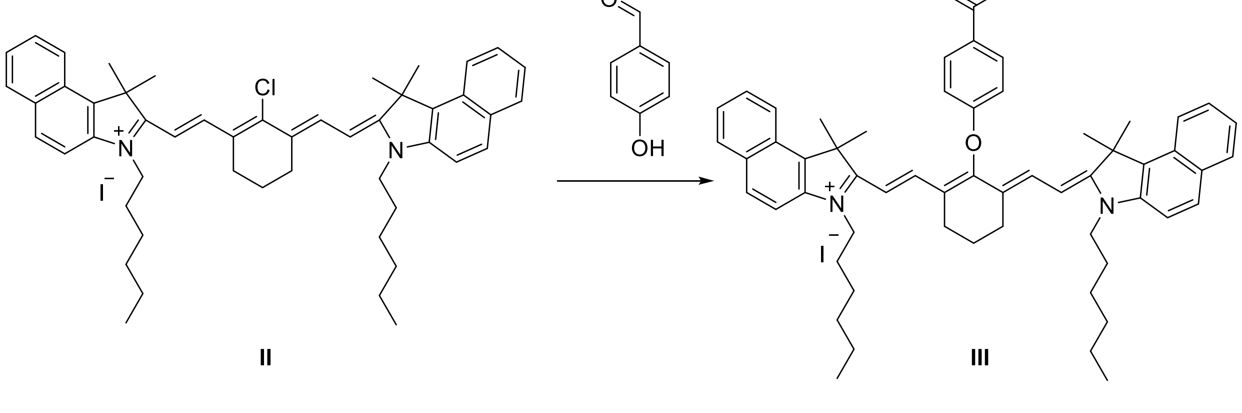

**II** **III**

$^{1}$H and $^{13}$C NMR spectra were recorded on Bruker Avance 400 (400 and 100 MHz, respectively) and Bruker Avance 600 (600 and 150 MHz). Residual signals of solvents were used as a reference (1H: $CDCl_3$, δ 7.26; $CD_2Cl_2$, δ 5.32. 13C: $CDCl_3$, δ 77.1, $CD_2Cl_2$, δ 54.0).

IR spectra were recorded on a Nicolet IR 200 Fourier-transform spectrometer (Thermo Scientific) using an internal reflectance accessory with a ZnSe attenuated total reflection (ATR) element with an incidence angle of 45°. The resolution was 4 $cm^{-1}$, the number of scans was 20.

High resolution mass spectra (HRMS) were recorded on an Agilent LC/MSD 1100 SL instrument with atmospheric pressure electrospray ionization (AP-ESI) in the positive ion detection mode (ion trap mass analyzer). Registration conditions: the nebulizer gas temperature (nitrogen) 300 °C at a rate of 12 L $min^{-1}$, the source potential 5000 V, the capillary outlet potential 150 V, solvent acetonitrile.

Reaction progress and purity of chromatographically separated compounds were monitored by thin-layer chromatography on Silica gel 60 F254 plates (Merck).

Chromatographic separation was carried out on a flash chromatography system Biotage Isolera Prime and on a column with MN Kieselgel 60 silica gel, 0.04—0.063 mm (230-400 mesh) ASTM and Interchim puriflash 60 si hp, 50 μm particle size.

**2-((E)-2-((E)-2-chloro-3-((E)-2-(3-hexyl-1,1-dimethyl-1,3-dihydro-2H-benzo[e]indol-2-ylidene)ethylidene)cyclohex-1-en-1-yl)vinyl)-3-hexyl-1,1-dimethyl-1H-benzo[e]indol-3-ium iodide (**compound **II)**

A mixture of 3-hexyl-1,1,2-trimethyl-1H-benzo[e]indol-3-ium iodide (0.50 g, 1.18 mmol), (E)-2-chloro-3-(hydroxymethylene)cyclohex-1-ene-1-carbaldehyde (0.10 g, 0.59 mmol) and (0.12 g, 1.42 mmol) of sodium acetate in 4 mL of ethanol was stirred at 50 °C for 5 h. After cooling to room temperature, an excess of diethyl ether was added, the precipitate was filtered off, washed on the filter with diethyl ether and dried. The dye was purified by column chromatography on silica gel in a mixture of eluents $CH_2Cl_2$: $CH_3OH$ (15:1, v/v) ($R_f$ = 0.59).

The yield was 0.290 g (69 %). $\lambda_{abs}$ = 824 nm (EtOH), $\lambda_{fl}$ = 834 nm (EtOH), $\varepsilon = 2.9 \cdot 10^5$ L $mol^{-1}$ $cm^{-1}$.

**1H NMR** (400 MHz, $CDCl_3$, δ, ppm, J/Hz): 0.90 (t, $^3J_{HH}$ = 7.09, 6H, $2CH_3$), 1.28 - 1.43 (m, 8H, $4CH_2$), 1.45 - 1.55 (m, 4H, $2CH_2$), 1.92 (quin, $^3J_{HH}$ = 7.47, 4H, $2CH_2$), 2.04 (s, 14H, $2C(CH_3)_2$, $CH_2$), 2.79 (t, $^3J_{HH}$ = 5.99, 4H, $CH_2SO_3$), 4.34 (t, $^3J_{HH}$ = 7.40, 4H, $2NCH_2$), 6.28 (d, $^3J_{HH}$ = 14.12, 2H, -CH=), 7.41 - 7.53 (m, 4H, Ar), 7.58 - 7.67 (m, 2H, Ar), 7.96 (d, $^3J_{HH}$ = 8.99, 4H, Ar), 8.14 (d, $^3J_{HH}$ = 8.56, 2H, -CH=), 8.45 (d, $^3J_{HH}$ = 14.37, 2H, -CH=).

**$^{13}$C NMR** (100 MHz, $CDCl_3$ δ, ppm, J/Hz): 13.62 ($CH_3$), 20.40, 20.73 (both $CH_2$), 22.09 ($CH_3$), 24.27, 26.27, 26.35, 27.30, 31.08 (all $CH_2$), 44.84 ($\underline{C}(CH_3)_2$), 50.75 ($^+NCH_2$), 100.57, 121.71, 124.78, 126.79, 127.42, 127.73, 130.42, 131.54, 139.29, 142.85 (all Ar), 110.49 (-CH=), 125.32 (-$\underline{C}(CH_2)$=), 129.79 (-$\underline{C}(CH_2)$=), 133.47 (C-Cl), 149.36 (-CH=), 173.22 (=C-N, $C=N^+$).

**HRMS-ESI**: m/z [M+] calculated for $C_{50}H_{60}N_2Cl$ 723.4440, found 723.4431.

**IR**, ν/$cm^{-1}$: 1474.8 (N=C-CH=), 1544.7 (C=C-N).

**2-((E)-2-((E)-2-(4-formylphenoxy)-3-((E)-2-(3-hexyl-1,1-dimethyl-1,3-dihydro-2H-benzo[e]indol-2-ylidene)ethylidene)cyclohex-1-en-1-yl)vinyl)-3-hexyl-1,1-dimethyl-1H-benzo[e]indol-3-ium iodide (**compound **III =** dye **2)**

A mixture of freshly prepared $CH_3ONa$ 0.029 g (0.54 mmol, 3 eq.) and para-hydroxybenzaldehyde 0.020 g (0.18 mmol, 1 eq.) was dissolved in a minimum amount of methanol. The solution was stirred at room temperature for 15 min. Then the solvent was evaporated and a light-yellow residue was formed. The resulting sodium 4-formylphenoate dissolved in 2 mL of DMFA was added to 0.150 g of **compound II** (0.18 mmol, 1 eq.) dissolved in 2 mL of DMFA in an argon atmosphere. The mixture was stirred for 4 h at room temperature, and DMFA was evaporated at reduced pressure. The obtained **compound III** was purified by column chromatography on silica gel in a mixture of eluents $CH_2Cl_2$:$CH_3OH$ (20:1, v/v), $R_f$ = 0.48. The isolated fraction was washed with diethyl ether.

The yield was 0.032 g (19 %). $\lambda_{abs}$ = 815 nm (EtOH), $\lambda_{fl}$ = 831 nm (EtOH), $\varepsilon = 4.3\cdot10^5$ L mol$^{-1}$ cm$^{-1}$.

**$^1$H NMR** (600 MHz, $CD_2Cl_2$, δ, ppm, J/Hz): 0.91 (t, $^3J_{HH}$ = 7.06, 6H, $2CH_3$), 1.32 - 1.40 (m, 8H, $4CH_2$), 1.46 (quin, $^3J_{HH}$ = 7.29, 4H, $2CH_2$), 1.64 (s, 12H, $4CH_3$), 1.85 (quin, $^3J_{HH}$ = 7.50, 4H, $2CH_2$), 2.11 (quin, $^3J_{HH}$ = 6.03, 2H, $CH_2$), 2.78 (t, $^3J_{HH}$ = 6.05, 4H, $2CH_2SO_3$), 4.13 (t, $^3J_{HH}$ = 7.57, 4H, $2NCH_2$), 6.09 (d, $^3J_{HH}$ = 14.21, 2H, -CH=), 7.34 (d, $^3J_{HH}$ = 8.53, 2H, Ar), 7.39 (d, $^3J_{HH}$ = 8.71, 2H, Ar), 7.47 (t, $^3J_{HH}$ = 7.34, 2H, Ar), 7.59 (t, $^3J_{HH}$ = 7.57, 2H, Ar), 7.92 - 7.98 (m, 6H, Ar), 8.01 (t, $^3J_{HH}$ = 8.48, 4H, Ar), 9.94 (s, 1H, CHO).

**$^{13}$C NMR** (151 MHz, $CD_2Cl_2$ δ, ppm, J/Hz): 14.28 ($2CH_3$), 21.69 ($CH_2$), 23.02 ($2CH_2$), 24.93 ($CH_2$), 27.12 ($2CH_2$), 27.81 ($4CH_3$), 27.99 ($2\underline{C}(CH_3)_2$), 31.95 ($2CH_2$), 45.30 ($2CH_2$), 51.42 ($2^+NCH_2$), 100.22 (2CH), 111.22 (Ar), 115.97, ($\underline{C}$=C(O)-$\underline{C}$), 121.83, 122.65, 125.68, 128.26, 128.55, 130.54, 131.16 (all Ar), 132.02 ($\underline{C}$-CHO), 132.44, 133.12, 134.45, 140.07 (all Ar), 141.14 (2CH), 162.82, 164.53 (both C=$\underline{C}$(O)-C), 174.13 (2C=N), 190.92 (CHO).

**HRMS-ESI**: m/z [M+] calculated for $C_{57}H_{65}N_2O_2$ 809.5041, found 809.5034.

**IR,** ν/cm$^{-1}$: 1468.05 (N=C-CH=), 1557.72 (C=C-N), 1693.68 (C=O).

**Table S1.** An example of a data table containing intensities of photographic images of samples listed in column A. Rows correspond to observations (wells of the plate) and columns correspond to different reaction times and different color channels (R – red, G – green) of the indicator reaction of Dye 1 with nitric acid. Only a part of the table is shown

| | A | B | C | D | E | F | G | H | I | J | K |
|---|---|---|---|---|---|---|---|---|---|---|---|
| 1 | | Dye 1 + HNO3 | | | | | | | | | |
| 2 | | R | | | | | G | | | | |
| 3 | | 0.5 min | 15 min | 21 min | 27 min | 43 min | 0.5 min | 15 min | 21 min | 27 min | 43 min |
| 4 | SRS 5w40 | 135,713 | 150,842 | 147,679 | 175,314 | 175,157 | 132,151 | 134,694 | 129,699 | 154,495 | 160,857 |
| 5 | SRS 5w40 | 132,526 | 144,355 | 141,596 | 173,061 | 173,072 | 133,153 | 129,146 | 123,963 | 151,526 | 157,395 |
| 6 | SRS 5w40 | 132,363 | 158,383 | 156,282 | 186,098 | 177,654 | 124,332 | 141,127 | 138,217 | 165,483 | 161,986 |
| 7 | SRS 5w40 | 122,32 | 150,458 | 144,808 | 172,901 | 174,417 | 118,404 | 132,879 | 127,149 | 151,143 | 159,869 |
| 8 | SRS 5w40 | 121,722 | 140,046 | 133,766 | 160,03 | 167,888 | 126,438 | 129,053 | 120,002 | 139,309 | 154,212 |
| 9 | SRS 5w40 | 128,203 | 145,791 | 139,29 | 166,409 | 173,949 | 129,17 | 131,847 | 122,905 | 143,88 | 159,886 |
| 10 | SRS 5w30 | 125,695 | 138,647 | 130,819 | 153,789 | 166,851 | 128,009 | 126,264 | 117,38 | 137,374 | 154,112 |
| 11 | SRS 5w30 | 129,276 | 146,664 | 140,788 | 173,618 | 172,244 | 128,387 | 131,75 | 124,32 | 154,582 | 159,025 |
| 12 | SRS 5w30 | 123,584 | 145,119 | 140,025 | 174,279 | 170,588 | 123,424 | 130,104 | 124,059 | 154,788 | 158,498 |
| 13 | SRS 5w30 | 115,774 | 143,853 | 139,659 | 171,273 | 166,657 | 112,618 | 127,961 | 123,201 | 152,089 | 154,514 |
| 14 | SRS 5w30 | 121,849 | 145,364 | 140,691 | 173,759 | 168,583 | 122,536 | 131,313 | 125,297 | 154,77 | 157,026 |
| 15 | SRS 5w30 | 123,416 | 140,864 | 128,085 | 168,079 | 167,391 | 126,161 | 129,139 | 115,041 | 147,803 | 156,164 |
| 16 | SRS 10w40 | 120,066 | 135,508 | 133,94 | 156,913 | 168,478 | 121,725 | 123,583 | 119,041 | 143,318 | 159,551 |
| 17 | SRS 10w40 | 122,42 | 130,316 | 127,544 | 157,861 | 164,513 | 127,297 | 121,103 | 114,211 | 141,582 | 154,422 |
| 18 | SRS 10w40 | 123,853 | 152,381 | 148,142 | 181,013 | 164,53 | 118,806 | 140,3 | 135,977 | 169,283 | 155,553 |
| 19 | SRS 10w40 | 117,8 | 145,689 | 140,797 | 170,331 | 161,884 | 116,841 | 133,039 | 127,49 | 156,77 | 152,861 |
| 20 | SRS 10w40 | 114,769 | 143,157 | 138,064 | 169,466 | 162,882 | 113,632 | 130,318 | 124,693 | 154,541 | 153,047 |
| 21 | SRS 10w40 | 119,003 | 135,193 | 121,366 | 164,286 | 162,517 | 122,684 | 125,073 | 110,267 | 148,872 | 153,128 |
| 22 | LUK | 119,237 | 125,817 | 130,438 | 153,256 | 164,447 | 121,882 | 115,313 | 114,384 | 138,253 | 151,635 |
| 23 | LUK | 110,676 | 128,899 | 151,043 | 176,422 | 166,04 | 114,811 | 116,897 | 138,521 | 164,217 | 156,076 |
| 24 | LUK | 109,357 | 124,425 | 126,453 | 150,546 | 156,26 | 112,433 | 114,703 | 111,145 | 134,519 | 143,407 |
| 25 | LUK | 105,979 | 128,676 | 129,553 | 152,247 | 155,453 | 107,074 | 117,619 | 114,543 | 137,297 | 143,766 |
| 26 | LUK | 111,962 | 132,888 | 128,655 | 159,679 | 161,457 | 114,633 | 121,747 | 114,802 | 144,018 | 150,268 |
| 27 | LUK | 114,844 | 125,513 | 109,498 | 157,12 | 159,171 | 117,962 | 116,826 | 98,648 | 141,213 | 147,868 |
| 28 | GAZ | 116,242 | 120,99 | 126,277 | 149,774 | 163,898 | 123,341 | 114,596 | 114,687 | 138,463 | 156,996 |
| 29 | GAZ | 115,961 | 120,786 | 127,532 | 149,515 | 159,142 | 122,981 | 113,652 | 114,947 | 138,119 | 152,17 |
| 30 | GAZ | 109,59 | 133,947 | 138,729 | 165,709 | 156,201 | 110,622 | 126,25 | 128,724 | 158,489 | 150,923 |
| 31 | GAZ | 105,421 | 128,878 | 130,153 | 153,64 | 153,845 | 112,44 | 120,269 | 119,095 | 143,344 | 148,28 |
| 32 | GAZ | 117,987 | 134,097 | 125,917 | 155,571 | 158,32 | 121,2 | 125,529 | 117,585 | 147,803 | 153,397 |
| 33 | GAZ | 116,535 | 116,417 | 106,407 | 153,599 | 158,843 | 123,469 | 112,214 | 100,472 | 144,347 | 154,115 |

**Table S2**. An example of a confusion matrix created by the XLSTAT LDA software for evaluating the accuracy of discrimination by a validation procedure

| True value | Predicted value | | | | | | Total number | % accuracy |
|---|---|---|---|---|---|---|---|---|
| | EVE | GAZ | LUK | SRS 10w40 | SRS 5w30 | SRS 5w40 | | |
| EVE | 0 | 0 | 0 | 0 | 0 | 0 | 0 | – |
| GAZ | 0 | 0 | 0 | 0 | 0 | 0 | 0 | – |
| LUK | 0 | 0 | **2** | 0 | 0 | 0 | 2 | 100.0% |
| SRS 10w40 | 0 | 0 | 0 | **2** | **1** | 0 | 3 | 66.7% |
| SRS 5w30 | 0 | 0 | 0 | 0 | 0 | 0 | 0 | – |
| SRS 5w40 | 0 | 0 | 0 | 0 | 0 | **1** | 1 | 100.0% |
| **Total** | 0 | 0 | 2 | 2 | 1 | 1 | **6** | **83.3%** |

*Note*. Six randomly selected observations were used as validation set. Accuracy was calculated as the number of correctly assigned observations to the total number of observations in the validation set (in this example, it was 5 / 6 = 0.833). This table is the result of one run of the software. The accuracy was calculated as the average of at least five runs, each time with another validation set.

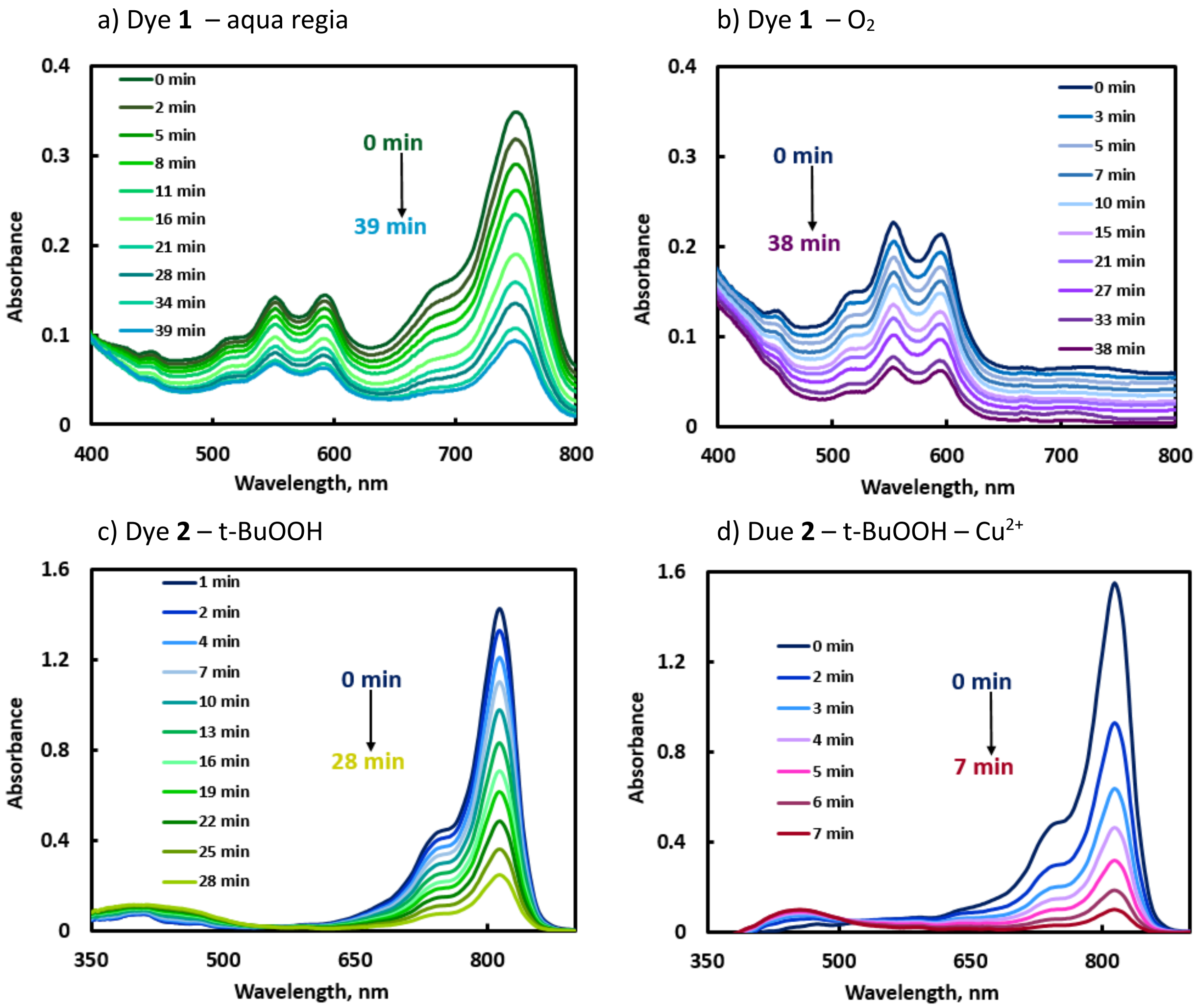


**Figure S1.** Absorbance spectra at different reaction time points for the indicator reactions named above the images. The reactant amounts were as follows: a) 20 µL of 0.1 g/L dye **1**, 560 µL of EtOH, 4 µL of aqua regia; b) 20 µL of 0.1 g/L dye **1**, 440 µL of EtOH, 120 µL of concentrated HCl; c) 20 µL of 0.1 g/L dye **2**, 760 µL of EtOH, 40 µL of 1 M HCl, 80 µL of 0.5 M t-BuOOH; d) 20 µL of 0.1 g/L dye **2**, 760 µL of EtOH, 40 µL of 1 M HCl, 40 µL $10^{-4}$ M $Cu^{2+}$, 60 µL of 0.5 M t-BuOOH.

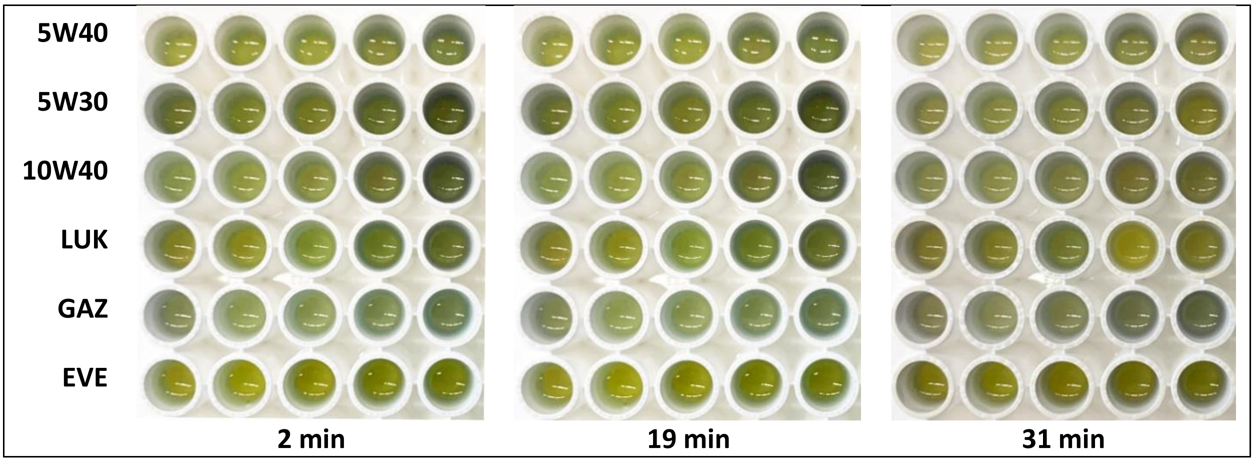


**Figure S2.** Visible-light images of the reacting system *dye **2** + $HNO_3$*, as captured by a smartphone camera. The replicate experiments for the same sample are in the row. Time after the reaction start is shown under each image.

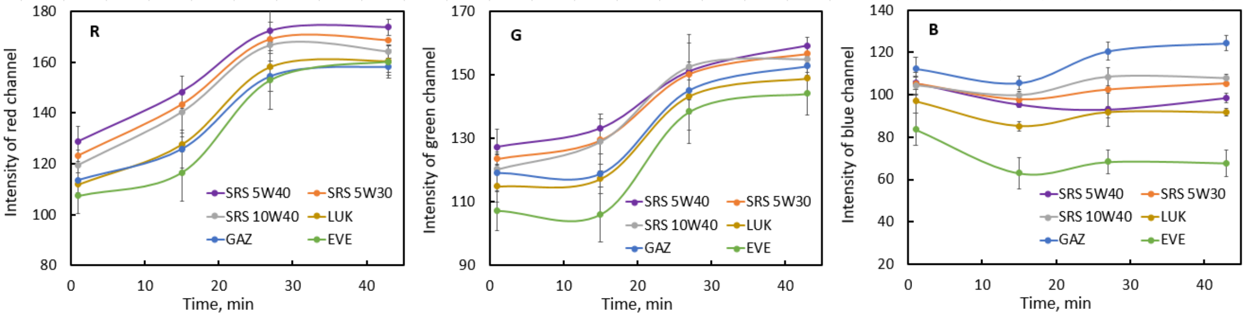


**Figure S3.** Kinetic curves for the reaction between dye **1** and $HNO_3$, as plotted for three color channels (R, G, and B). The results were averaged for the 6 parallel runs of each sample shown in the legend. The error bars represent a standard deviation.

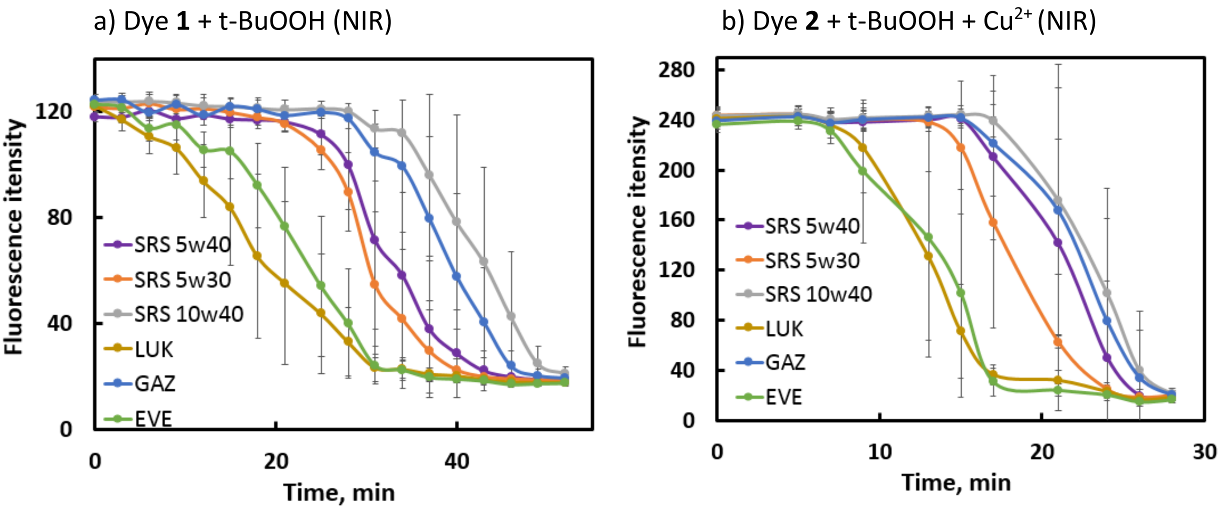


**Figure S4.** Kinetic curves of indicator reactions: (a) Dye **1** + t-BuOOH and (b) Dye **2** + t-BuOOH + $Cu^{2+}$ in the presence of oil samples shown in the legend. Each curve is a result of averaging of 6 replicate runs, and the error bars correspond to the standard deviations. Ordinate is the average intensity for the near-IR photographs.

**Accuracy of assignment of an oil sample as a whole** (six observations) based on the known accuracy of assignment of a single observation (as received from LDA or kNN techniques)

In this example, let the accuracy of a single observation be 93%, the general sample be $N$ = 1000 observations, that contains $K$ = 930 correct observations and $N - K$ = 70 incorrectly assigned observations. The whole sample of $n$ = 6 observations will be recognized incorrectly if $k$ = 3, 4, 5 or 6 out of 6 observations are incorrect. Let us calculate these probabilities using a known formula (https://www.matburo.ru/tvart_sub.php?p=calc_gg_item, accessed on July 16th, 2023):

$$P = \frac{C_K^k \cdot C_{N-K}^{n-k}}{C_N^n}$$

Here $P$ is the target probability of the incorrect assignment and $C_K^k$ is the number of combinations of $k$ in $K$ that equals $C_K^k = \frac{K!}{k!(K-k)!}$ (in different countries the notations may vary: https://en.wikipedia.org/wiki/Combination)

We have to sequentially calculate the probabilities of incorrect assignment for $k$ = 3, 4, 5 or 6 out of 6 observations and sum them up. The calculation was performed using the online service provided by the website mentioned above. For $k$ = 3 we obtained:

$$P_3 = \frac{C_{930}^3 \cdot C_{70}^3}{C_{1000}^6} = 0.00534$$

Similarly, for $k$ = 4 we obtained $P_4$ = 0.00028 and for $k$ = 5, $P_5$ = 0.00000. Therefore, the overall probability will be $P = P_3 + P_4$ = 0.0056, or 0.56%. So, the accuracy of discrimination of the whole sample of 6 observations will be **99.44%**. Using the provided formulas or the online service, the accuracies for other conditions can be easily obtained.

## Reference

[1] E. Lima, A.G. Barroso, M.A. Sousa, O. Ferreira, R.E. Boto, J.R. Fernandes, P. Almeida, S.M. Silvestre, Ad.O. Santos, L.V. Reis, Picolylamine-functionalized benz[e]indole squaraine dyes: Synthetic approach, characterization and in vitro efficacy as potential anticancer phototherapeutic agents, *Eur. J. Med. Chem.* **229** (2022) 114071. https://doi.org/10.1016/j.ejmech.2021.114071.
